# Editorial: Artificial intelligence applications in chronic ocular diseases, volume II

**DOI:** 10.3389/fcell.2026.1762437

**Published:** 2026-02-24

**Authors:** Weihua Yang, Huihui Fang, Yanwu Xu, Wei Chi

**Affiliations:** 1 Shenzhen Eye Hospital, Shenzhen Eye Medical Center, Southern Medical University, Shenzhen, China; 2 Pazhou Lab, Guangzhou, Guangdong, China; 3 School of Future Technology, South China University of Technology, Guangzhou, Guangdong, China

**Keywords:** artificial intelligence in ophthalmology, chronic ocular diseases, clinical workflow integration, multimodal imaging and analysis, ocular biomarkers and systemic diseases

## 1 Introduction

Artificial intelligence (AI) has rapidly reshaped the landscape of ophthalmic research and clinical practice, offering unprecedented capabilities for disease detection, quantitative phenotyping, risk prediction and decision support across a broad spectrum of chronic ocular conditions (Xu and Yang, 2023; Wang et al., 2024). In recent years, advances in deep learning, multimodal fusion and large-scale representation learning have driven substantial progress in the diagnosis and screening of retinal diseases such as diabetic retinopathy (DR) (Li et al., 2023; Ming and Lei, 2019), macular disorders (Feng et al., 2023) and high myopia–related pathology (Wu et al., 2022), as well as in glaucoma (Li et al., 2023; Cong et al., 2024; Qian et al., 2024), cataract, anterior segment abnormalities (Jiang et al., 2023), ocular surface disease (Ji et al., 2022; Mimazhuoma and Ji, 2023) and orbital disorders.

Parallel to these developments, the eye has increasingly been recognized as a sensitive biomarker source for systemic chronic diseases—including cardiovascular disease, hypertension, diabetes and peripheral neuropathy—supported by growing evidence that ocular microvascular, neurostructural and biomechanical features reflect systemic pathophysiology (Hui et al., 2024; Liu Z. et al., 2025). Together, these developments underscore the dual role of ophthalmic AI as both a disease-focused diagnostic tool and a window into whole-body health.

At the same time, methodological advances in quantitative image analysis (Zhou et al., 2023), 3D segmentation (Wu et al., 2024; Liu Z. et al., 2025), cross-device standardization, quality assurance and multimodal intelligent systems have transformed AI from a standalone algorithmic tool into a foundational analytical infrastructure (Wu et al., 2025) capable of integrating imaging, clinical data and text-based information. AI is now increasingly embedded within clinical workflows (Li et al., 2024), supporting perioperative decision-making for glaucoma, refractive and cataract surgeries, predicting anatomical and functional treatment responses, and enabling long-term monitoring in chronic disease management (Yang et al., 2024).

The integration of multi-omics modalities—such as aqueous humor proteomics, transcriptomics and functional neuroimaging—further extends AI applications from phenotype recognition to mechanistic discovery, offering insights into aging, neurovascular coupling and immunometabolic remodeling in chronic ocular conditions. Meanwhile, systematic reviews and population-level studies emphasize the potential of AI to enhance public health strategies, including large-scale screening programs, disease surveillance, teleophthalmology and medical education (Gong et al., 2024).

Despite these promising advances, significant challenges remain in the areas of generalizability, robustness to real-world variability (Liu M. et al., 2025), interpretability, multimodal data integration, regulatory readiness and ethical governance (Li et al., 2024; Gong et al., 2024). These limitations highlight the need for foundation models (Wu et al., 2025), cross-organ risk assessment platforms, causality-aware and transparent algorithms, and standardized evaluation frameworks that can support safe and equitable deployment across diverse populations and healthcare environments.

We launched a new round of this research initiative to systematically explore the latest advancements in artificial intelligence applications for chronic ocular diseases. This year, we received 82 submissions, and after rigorous peer review and quality assessment, 57 representative papers were accepted for publication. To date, these articles have accumulated over 28,000 downloads, with a combined 129,000 views and downloads, reflecting the sustained interest in and significant scientific impact of AI-driven research in the field of chronic eye diseases.

**FIGURE 1:**
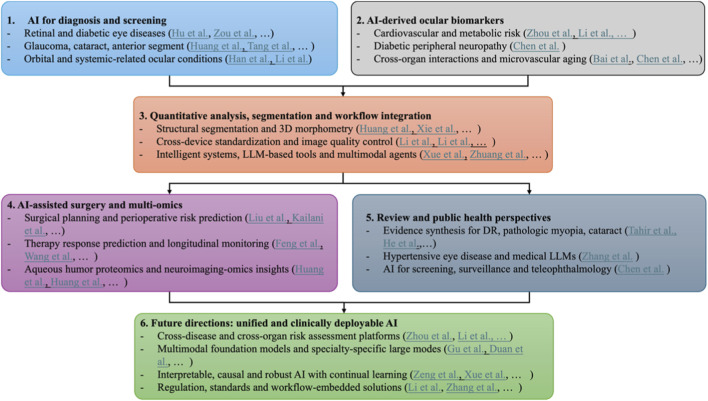
Logical relationships among major AI research directions in chronic ocular diseases. Disease‐focused and biomarker‐focused studies (Ch. 2–3) feed into a shared infrastructure of quantitative analysis and workflow integration (Ch. 4), which branches into surgical/precision medicine and public health applications (Ch. 5–6) and jointly informs future unified and clinically deployable AI ecosystems (Ch. 7).

This editorial provides a comprehensive synthesis of these AI applications in chronic ocular diseases across six major domains—disease diagnosis and screening, ocular biomarkers of systemic disease, quantitative analytics and workflow integration, AI-assisted surgery and treatment planning, public-health and review perspectives, and future research directions. The conceptual relationships among these domains are summarized in Figure 1, illustrating how disease-oriented and biomarker-driven research converges into shared analytical infrastructures and expands toward surgical, public-health and unified ophthalmic AI ecosystems.

## 2 AI for diagnosis and screening of chronic ocular diseases

### 2.1 Retinal and diabetic eye diseases

In the field of diabetic retinopathy (DR) and related retinal diseases, multiple studies have systematically demonstrated the potential of AI across the entire pipeline from lesion detection through microcirculation assessment to risk stratification.

At the image level, Hu et al. introduced a lightweight attention mechanism based on ultra-widefield (UWF) fundus images, achieving substantial improvements in DR lesion detection accuracy while maintaining low computational complexity. This work highlights the engineering advantage of “high performance + low computational cost” in resource-constrained environments. Zuo et al. proposed a multi-scale visual Mamba (MRVM) model on optical coherence tomography (OCT), achieving classification accuracies of approximately 98.98% and 96.21% on two public retinal datasets, demonstrating the clear advantages of next-generation sequence modeling architectures in interpreting volumetric retinal data. For color fundus photography, Zhang et al. fused deep features extracted by convolutional neural networks (CNNs) with radiomics features, and improved DR classification performance using a combination of label smoothing and graph-constrained collaborative learning, emphasizing the role of “multi-feature fusion + regularization” in suppressing overfitting and enhancing inter-class separability.

In the OCT angiography (OCTA) domain, Wu et al. used swept-source OCTA (SS-OCTA) and found that the choroidal vascularity index (CVI) in highly myopic patients with diabetes was significantly lower than in patients with DR alone, suggesting that CVI, as a quantitative AI-derived biomarker, may be useful for early risk stratification in high-risk populations. Li et al. examined peripapillary atrophy (PPA) subregions with SS-OCTA and found that γ-zone PPA was associated with a reduced risk of DR. They proposed that “myopia-related posterior pole thinning/microvascular depletion” may exert a structural “protective effect” against DR, providing new imaging evidence for the interaction between myopia and DR.

For multiple retinal diseases, the WARN model proposed by Guo et al. achieved strong performance in classifying seven common retinal conditions, while Qi et al. reported similarly robust discriminative performance, further underscoring the practical value of AI in multi-disease retinal diagnosis. In terms of multimodal fusion, Gu et al. developed the Fusion-MIL model that jointly models color fundus photographs and OCT representations, demonstrating superior diagnostic performance over any single-modality model and strong cross-device generalizability and fine-grained grading capacity—features particularly valuable for long-term follow-up in multicenter, multi-device environments. On UWF images, Duan et al. built a model capable of automatic recognition and classification of multiple ocular diseases, providing a technical foundation for optimizing outpatient workflows and automated pre-screening. For fluorescein fundus angiography (FFA), Duan et al. further showed that knowledge-enhanced pretraining could markedly improve diagnostic accuracy and generalization, demonstrating that domain knowledge injection is particularly beneficial for complex multi-frame imaging.

Overall, this body of work indicates that AI in retinal and DR applications has evolved from single-disease, single-modality classifiers toward multi-disease, multimodal and multi-scenario “system-level risk assessors”.

### 2.2 Glaucoma, cataract and anterior segment disorders

In glaucoma, Huang et al. constructed a random forest model guided by Boruta feature selection using demographic characteristics, metabolic indicators and biochemical parameters to predict the risk of neovascular glaucoma (NVG) in patients with proliferative diabetic retinopathy (PDR). This study highlights the value of cross-system modeling that integrates “internal medicine metabolic indicators + ophthalmic imaging/clinical data”, and shows that traditional machine learning models remain highly interpretable and practical in scenarios with limited sample sizes.

In cataract, Tang et al. built an AI model using slit-lamp retroillumination images to achieve automatic diagnosis and grading, establishing a novel quantitative assessment system that can significantly improve efficiency and consistency in primary care and large-scale screening.

Anterior segment and ocular surface diseases are also key components of chronic ocular disease management. Wang et al. analyzed meibomian gland energy curves derived from upper eyelid infrared imaging to quantify structural changes associated with Demodex infestation, and proposed that the derived parameters can serve as early, non-invasive biomarkers for dry eye and chronic ocular surface inflammation, thus providing a new “structure–function” quantitative pathway.

### 2.3 Orbital and systemic-related ocular conditions

For orbital diseases, Han et al. developed a deep learning model for diagnosing thyroid-associated ophthalmopathy (TED) based on facial photographs and clinical records. The model achieved high accuracy for key phenotypes such as inflammation associated with clinical activity score (CAS), eyelid retraction and motility restriction, and emphasized that visual explanations are critical for supporting individualized treatment planning. In traumatic orbital fractures, Li et al. built an AI-assisted diagnostic and treatment decision system using CT images, which improved the accuracy of detecting trapdoor fractures and reduced surgical complications, thereby laying the groundwork for future intelligent surgical robots and intraoperative navigation systems.

In summary, this chapter provides a panoramic view of AI in the diagnosis and screening of chronic ocular diseases, illustrating a cross-disease, multimodal technological progression from retinal diseases to glaucoma, cataract, ocular surface and orbital disorders.

## 3 AI-derived ocular biomarkers and systemic chronic diseases

The eye is often described as a “window to systemic diseases”. Numerous studies have focused on extracting ocular imaging biomarkers related to cardiovascular and metabolic conditions.

### 3.1 Cardiovascular and metabolic diseases

In coronary artery disease (CAD), Zhou et al. used OCTA to derive quantitative retinal microvascular features and developed a CAD-assisted diagnostic model, demonstrating that retinal OCTA biomarkers can serve as standardized tools for early CAD screening and support cardiology decision-making. Regarding hypertensive eye disease, Li et al. conducted a systematic review summarizing deep learning models based on fundus photographs for early hypertension screening and risk stratification, while highlighting key limitations in model generalizability, robustness to low-quality images and interpretability. Ding et al. further leveraged deep learning to extract features from OCTA, more precisely characterizing hypertension-related changes in retinal vascular morphology and perfusion, and showing that AI has considerable potential as a high-throughput screening tool for hypertension and its complications.

### 3.2 Diabetic peripheral neuropathy

In diabetic peripheral neuropathy (DPN), Chen et al. used corneal confocal microscopy (CCM) images to compare transformer-based and CNN-based networks for DPN binary classification, and found that a transformer-based DLA exhibited higher potential for rapid screening. Such work brings “ocular micro-neural structures” into AI pipelines for systemic diabetes management and represents an important component in constructing lifelong disease trajectories.

### 3.3 Cross-organ interactions and microvascular aging

In terms of retinal vasculature and optic nerve head morphology, Bai et al. compared populations with high *versus* low cerebro-cardiovascular risk and found significant differences in vascular complexity and cup-to-disc area ratio, providing ocular imaging evidence for cardiovascular risk stratification. In a cohort of patients with type 2 diabetes mellitus (T2DM), Chen et al. reported that specific retinal vascular parameters—such as mean branch segment length and vessel density—were significantly associated with mild-to-moderate non-proliferative DR (NPDR), suggesting that these features may serve as preclinical biomarkers of DR-related microvascular abnormalities.

From the perspective of hemodynamics and vessel wall structure, Jiang et al. used spectral-domain OCT (SD-OCT) and the full-width at half maximum (FWHM) method to noninvasively measure a series of parameters (including RALD, RAOD, RVLD, RVOD, AWT, VWT and AVR), and demonstrated that they were significantly associated with internal carotid artery stenosis. In another study, Jiang et al. applied OCTA and PKSEA-Net to analyze retinal microvascular morphology in individuals with gestational diabetes mellitus (GDM), suggesting that AI tools could enable early detection of microvascular alterations in high-risk pregnant populations.

In addition, Wei et al. used intelligent quantitative algorithms and found that allergic conjunctivitis significantly altered meibomian gland length and central morphology, providing quantifiable structural indicators for chronic ocular surface inflammation. Hao et al. combined pattern visual evoked potentials (PVEP) with machine learning to predict best-corrected visual acuity in patients with ocular trauma, establishing a pathway for coupling imaging and functional evaluation.

Taken together, this chapter extends the focus from local ocular phenotypes to “eye–heart–brain–metabolic” interactions, markedly enhancing the interdisciplinary depth of AI research in chronic ocular diseases.

## 4 Quantitative analysis, segmentation and clinical workflow integration

### 4.1 Structural segmentation and morphological quantification

In structural segmentation, Huang et al. designed a large-kernel multi-scale attention module for choroidal vessel segmentation on OCT, effectively addressing challenges such as low contrast and fuzzy boundaries. Based on the segmentation results, they performed three-dimensional reconstruction and morphometric analysis, revealing significant structural differences in the choroid between highly myopic and healthy eyes. Xie et al. proposed M3B-Net for retinal vessel segmentation on combined UWF and FFA images, introducing a selective fusion module (SFM), a local perception fusion module (LPFM) and an attention-guided upsampling module (AUM). The model significantly improved segmentation of fine vessels in ultra-widefield high-resolution images. Liu et al. validated DS2TUNet on multiple public datasets (DRIVE, CHASE_DB1, ROSE-1) and a clinical dataset of central serous chorioretinopathy (CSC), showing that the model achieved state-of-the-art or superior performance across multiple metrics and exhibited strong cross-dataset transferability.

These studies indicate that AI-driven fine-grained segmentation and 3D reconstruction have become essential technical supports for quantifying structural changes in chronic ocular diseases.

### 4.2 Clinical workflow integration and intelligent systems

For image quality control, Li et al. developed the DeepMonitoring system using corneal images acquired via smartphones. The system can both determine whether an image is of low quality and localize the source of the quality Research Topic, guiding operators to retake images when necessary and thus ensuring input quality for mobile ophthalmic AI systems. In real-world applications for chronic ocular diseases, such QA/QC is a prerequisite for stable model deployment.

In another study, Li et al. proposed the MOSAIC system, which integrates ocular surface images and textual information and demonstrates strong few-shot learning capability: even with limited training data, it can accurately manage ocular surface diseases. Its potential for deployment on mobile devices is particularly well aligned with the needs of primary care and resource-limited settings.

In multimodal question answering and large model applications, Xue et al. explored the use of ChatGPT for triaging ocular trauma in emergency settings, showing that it may improve preliminary assessment and triage efficiency but still exhibits clear limitations in understanding clinical images. Yang et al. showed that customized large language models (LLMs) can become effective educational tools for medical students and ophthalmologists. Zeng et al. incorporated ChatGPT into traditional teaching to enhance understanding of rare diseases such as retinitis pigmentosa (RP), demonstrating that AI as a knowledge-augmentation tool can complement conventional education and foreshadowing “hybrid AI-augmented teaching” paradigms.

With respect to system-level AI solutions, Zhuang et al. proposed a multimodal agent that illustrates both the feasibility and necessity of constructing specialized AI solutions for complex clinical scenarios.

Overall, this chapter illustrates how AI technologies have evolved from “research-grade models” to “systems that can be embedded into clinical workflows”, forming an end-to-end path from segmentation and parameter extraction to standardization, quality control and AI agents.

## 5 AI-assisted surgical decision-making, treatment planning and multi-omics insights

### 5.1 Surgical planning and perioperative risk prediction

Research on surgery and treatment demonstrates the value of AI throughout the perioperative cycle, from preoperative decision support through intraoperative safety management to postoperative prognostication.

In glaucoma surgery, Liu et al. showed that anatomical features of patients with primary angle-closure glaucoma (PACG) are major determinants of refractive instability after phacoemulsification with intraocular lens implantation (PE + IOL), based on preoperative refraction and ocular biometry. They recommended that axial length (AL), lens thickness (LT), white-to-white corneal diameter (WTW) and the AL/corneal radius (AL/CR) ratio should be jointly considered when selecting IOL power. In a systematic review, Kailani et al. concluded that existing AI models for glaucoma surgery prediction generally outperform traditional statistical methods, but are limited by lack of external validation and heterogeneous success criteria. They stressed the need for multicenter prospective studies and open datasets to improve model reliability.

In refractive and cataract surgery, Liu et al. demonstrated that ocular rotational magnitude in SMILE procedures is significantly associated with residual postoperative astigmatism, providing quantitative evidence to inform preoperative planning and rotational compensation strategies. Su et al. constructed a surgical decision model for highly myopic cataract based on slit-lamp images, OCT and biometry, achieving good performance in complex decision-making scenarios. Regarding intraoperative complications, Zhu et al. used preoperative panoramic corneal images and surgical videos to build models that predict OBL patterns and the risk of OBL formation during SMILE procedures. Both deep residual networks and GAN-based models proved effective, supporting preoperative risk stratification and intraoperative warning.

### 5.2 Treatment response prediction and longitudinal follow-up

For pharmacological treatment and prognostication, Feng et al. used OCT images to generate post–anti-VEGF treatment response images, demonstrating the potential of generative models in predicting treatment outcomes. Wang et al. employed SS-OCTA to evaluate retinal and choroidal hemodynamic changes in patients with atrial fibrillation after radiofrequency catheter ablation (RFCA). Changes in choroidal thickness and CVI were found to possibly reflect compensatory redistribution of cardiac output, suggesting that ocular microcirculation may serve as a sensitive biomarker for monitoring anti-arrhythmic therapy. Lu et al. combined OCT structural features with blood metabolic and hematologic indicators to build a model predicting anatomical response to anti-VEGF therapy in diabetic macular edema (DME), thereby enabling individualized treatment optimization.

In long-term monitoring of orthokeratology (ortho-K), Wu et al. analyzed blinking behavior in children using a deep learning system based on U-Net and Swin Transformer. They found that increased frequency of incomplete blinks was closely associated with reduced tear film stability, suggesting that blinking patterns can be an important monitoring indicator for long-term ortho-K wear.

Collectively, these studies demonstrate the system-level value of AI in “perioperative management” of chronic ocular diseases, forming a closed loop from precise preoperative assessment and intraoperative risk control to postoperative outcome prediction and functional monitoring.

### 5.3 Multi-omics-driven mechanistic studies

Multi-omics research provides a new dimension for understanding mechanisms and targeted interventions in chronic ocular diseases.

In aqueous humor proteomics, Huang et al. analyzed age-related changes in the aqueous humor proteome, identifying 179 significantly age-associated proteins and further selecting 11 characteristic proteins for aqueous age prediction and 22 potential regulators. Their findings highlighted oxidative damage, matrix dysregulation and protein homeostasis imbalance as hallmark signatures of ocular aging. Regarding central nervous system involvement in DR, Huang et al. were the first to integrate functional MRI (fMRI) with transcriptomics to elucidate the genetic determinants of disrupted interhemispheric connectivity in DR, pointing to the complex interplay of neurovascular, metabolic and neurodegenerative pathways as key contributors to DR-related cognitive and visual dysfunction.

In thyroid-associated ophthalmopathy, Shu et al. analyzed gene expression profiles of TED-related lacrimal gland hypertrophy and orbital fat expansion, revealing significant pathophysiological differences between these two manifestations and building a TED diagnostic prediction model based on KIAA0319 and PRDX4. This work provides both omics-level evidence and a modeling prototype for noninvasive, prospective TED diagnosis.

In summary, this chapter illustrates the expansion of AI from a “clinical imaging tool” to an integrated role in “surgical medicine, treatment response and mechanistic discovery”, serving as an important conceptual upgrade in the context of chronic ocular disease research.

## 6 Review and public health perspectives

Several systematic reviews and methodological studies provide a high-level perspective that underpins this narrative review.

For DR, the systematic review and meta-analysis by Tahir et al. showed that AI-assisted screening achieves higher sensitivity than human graders while maintaining comparable specificity, supporting the use of AI as a reliable alternative or adjunct for DR screening. Xu et al. systematically reviewed AI models for DR diagnosis and treatment from a methodological standpoint, discussing issues of bias, transparency and ethics, and outlining prospects for clinical translation.

For pathologic myopia and cataract management, He et al. and Lu et al. summarized the progress of AI in screening, grading and prognostic assessment, emphasizing the importance of AI for long-term management of progressive chronic eye diseases. Li et al.reviewed AI models based on fundus imaging for hypertensive eye disease, discussing their current status and limitations.

With respect to LLMs and multimodal medical foundation models, Zhang et al. outlined their potential roles in screening, decision-making and individualized treatment, and identified three major barriers to clinical adoption: difficulties in acquiring multimodal data, limited interpretability and the lack of standardized validation frameworks. From a public health perspective, Chen et al. systematically analyzed the value of AI in large-scale screening, disease surveillance, telemedicine and continuing education, highlighting the “amplifier” role of AI in strengthening ophthalmic public health capacity.

These review-oriented contributions furnish a robust evidence base for the overarching framework of “Artificial Intelligence Applications in Chronic Ocular Diseases” and empirically support the research directions summarized in this article.

## 7 Future directions: toward unified and clinically deployable AI for chronic ocular diseases

Based on the evidence above, future directions for AI in chronic ocular diseases can be summarized as follows.

### 7.1 From single-disease models to cross-disease, cross-organ unified risk assessment platforms

Existing work has extended beyond DR to encompass glaucoma, pathologic myopia, cataract, TED, dry eye and ocular surface disorders, while also identifying ocular biomarkers for systemic diseases such as CAD, hypertension and DPN (Zhou et al., Li et al., Ding et al., Chen et al.). It will be necessary to establish joint risk scoring systems covering the “eye–brain–heart–metabolic” axis and to realize multi-disease risk co-assessment and long-term follow-up within a unified feature space integrating phenotypes, omics and imaging.

### 7.2 Multimodal and multi-scale fusion: integrated modeling of imaging, text, omics and longitudinal data

From Fusion-MIL (Gu et al.) and knowledge-enhanced pretraining (Duan et al.) to the joint analysis of aqueous humor proteomics and fMRI–transcriptomics (Huang et al., Huang et al.), it is evident that future models should not be confined to a single modality. The development of multimodal models capable of simultaneously handling 2D/3D imaging, clinical text, omics data and longitudinal follow-up will be key to achieving a “vertical–horizontal integrated” panoramic view of chronic ocular disease.

### 7.3 Foundation models and specialty-specific multimodal large models

Current research has begun to explore GPT-like large language models (LLMs) in medical education (Zeng et al.), clinical question answering (Xue et al., Yang et al.) and multimodal decision support (Zhang et al., Zhuang et al., Li et al.). In the future, ophthalmology-focused multimodal foundation models could be developed using large-scale, unlabeled ophthalmic images, electronic medical records and omics data for self-supervised pretraining, followed by adaptation to multiple downstream tasks with limited labeled data. This would enable a new paradigm in which “one foundation model supports multiple tasks and scenarios”.

### 7.4 From black box to interpretability and controllability: trustworthy AI and causal inference

Many studies have underscored the lack of interpretability as a key obstacle to clinical deployment of AI (Li et al. , Zhang et al., Kailani et al.). Future work needs to incorporate attention visualization, inherently interpretable architectures, causal inference methods and uncertainty quantification into AI models for chronic ocular diseases, thereby enhancing transparency of the decision process and providing clinicians with traceable chains of evidence (for example, the specific vessel segments or choroidal layers contributing to a decision). This, in turn, will help generate new hypotheses about disease mechanisms.

### 7.5 Robustness and domain generalization in real-world settings

Real-world management of chronic ocular diseases is characterized by multiple centers, devices, ethnicities and coexisting diseases. The OCT platform conversion study by Tian et al., the image quality monitoring work by Li et al., and cross-dataset validation of segmentation models (Liu et al., Xie et al.) provide practical examples for improving robustness. Future research should systematically address domain generalization and domain adaptation, and build continually learning systems capable of online updating to cope with device upgrades, changes in imaging protocols and population heterogeneity.

### 7.6 From tools to decision-making loops: workflow-embedded AI solutions

From system-level tools such as MOSAIC and Fusion-MIL (Li et al., Gu et al.) to glaucoma and cataract surgical decision models (Liu et al., Su et al., Kailani et al.), AI is moving from a peripheral auxiliary role into central positions within clinical workflows. Future efforts should emphasize deep integration with hospital information systems (HIS), electronic medical records (EMR), surgical navigation and telemedicine platforms, forming a complete decision-making loop that spans “screening–diagnosis–surgery–follow-up–public health”.

### 7.7 Ethics, regulation and standardized evaluation frameworks

Review articles have consistently highlighted ethical, privacy and standardization gaps (Xu et al., Zhang et al., Chen et al., Kailani et al.). Because chronic ocular disease AI involves long-term follow-up and allocation of healthcare resources, it is especially prone to issues of fairness and accessibility. Future work should promote the development of data standards, model registration systems and tiered validation frameworks at international and national levels to ensure safety, fairness and sustainability of AI systems across regions and populations.

## 8 Conclusion

Artificial intelligence is rapidly transforming the landscape of chronic ocular disease research and clinical practice. Across diagnostic modeling, ocular biomarker discovery, quantitative analytics, surgical decision-making, public health applications and mechanistic multi-omics studies, the 57 accepted papers in this research topic collectively demonstrate the breadth and maturity of AI-driven innovation in ophthalmology. The evidence synthesized in this editorial illustrates how AI has evolved from single-modality diagnostic tools into integrated, multimodal ecosystems capable of supporting risk stratification, longitudinal monitoring, precision surgical planning and interdisciplinary insights linking ocular and systemic health. Despite these advances, major challenges persist—including limited generalizability across devices and populations, insufficient interpretability, heterogeneous validation standards and the absence of unified regulatory frameworks. Addressing these gaps will require the development of robust, transparent and clinically grounded AI systems, as well as large-scale multimodal datasets, specialty-specific foundation models and workflow-embedded decision-support pipelines. Looking ahead, the convergence of imaging, clinical data, omics and large language models promises to reshape the management of chronic ocular diseases, paving the way toward unified, equitable and clinically deployable AI platforms that can support lifelong ocular health.
